# Rational Design of Porous Poly(ethylene glycol) Films as a Matrix for ssDNA Immobilization and Hybridization

**DOI:** 10.3390/bioengineering9090414

**Published:** 2022-08-24

**Authors:** Zhiyong Zhao, Saunak Das, Michael Zharnikov

**Affiliations:** Angewandte Physikalische Chemie, Universität Heidelberg, 69120 Heidelberg, Germany

**Keywords:** poly(ethylene glycol), STAR-PEGs, ssDNA immobilization, ssDNA hybridization, electrochemical impedance spectroscopy, X-ray photoelectron spectroscopy

## Abstract

Poly(ethylene glycol) (PEG) films, fabricated by thermally induced crosslinking of amine- and epoxy-terminated four-arm STAR-PEG precursors, were used as porous and bioinert matrix for single-stranded DNA (ssDNA) immobilization and hybridization. The immobilization relied on the reaction between the amine groups in the films and N-hydroxy succinimide (NHS) ester groups of the NHS-ester-decorated ssDNA. Whereas the amount of reactive amine groups in the films with the standard 1:1 composition of the precursors turned out to be too low for efficient immobilization, it could be increased noticeably using an excess (2:1) concentration of the amine-terminated precursor. The respective films retained the bioinertness of the 1:1 prototype and could be successfully decorated with probe ssDNA, resulting in porous, 3D PEG-ssDNA sensing assemblies. These assemblies exhibited high selectivity with respect to the target ssDNA strands, with a hybridization efficiency of 78–89% for the matching sequences and full inertness for non-complementary strands. The respective strategy can be applied to the fabrication of DNA microarrays and DNA sensors. As a suitable transduction technique, requiring no ssDNA labeling and showing high sensitivity in the PEG-ssDNA case, electrochemical impedance spectroscopy is suggested.

## 1. Introduction

Immobilization of single-stranded DNA (ssDNA) on solid supports is an important issue in the physical chemistry of interfaces and biomedical research, widely applied in biological detection, microarray technology, and related fields [1,2,3]. Usually, ssDNA are immobilized on the target substrate by their decoration with a suitable anchoring group which has a strong affinity to the substrate. In particular, thiol-substituted ssDNA strands, capable of assembling on coinage-metal substrates (above all on gold), are frequently used [4,5,6,7]. Specifically modified ssDNA strands can also be attached to surfaces precoated with self-assembled monolayers (SAMs) [8,9] or polymers [10,11], relying on covalent bonding between specific docking groups of ssDNA and matching terminal groups of SAMs or polymers. 

To achieve a high hybridization efficiency and to form individual sensing spots, it is frequently necessary to immobilize ssDNA into a biocompatible matrix resisting non-specific ssDNA-surface interactions [12,13,14]. In this way, one can control the density of the immobilized probe ssDNA and suppress nonspecific adsorption of target ssDNA beyond the predefined sensing spots, thus improving the specificity and efficiency of a particular assembly or a device. The most frequently used bioinert material in this context is poly(ethylene glycol) (PEG). PEG is a hydrophilic polymer with remarkable biocompatible properties, prohibiting nonspecific adsorption of proteins, oligonucleotides, bacteria, and other bioorganisms [15,16,17,18,19]. This material is used in particular for the decoration of SAMs, in the form of oligo(ethylene glycol) (OEG) tail groups, resulting in the fabrication of bioinert surfaces and interfaces [20,21,22,23,24]. Properly decorated ssDNA can then be immobilized onto the respective supports, either as a component of binary ssDNA-OEG SAMs or within a predefined spot, using a lithographic approach [25,26]. 

**Figure 1:**
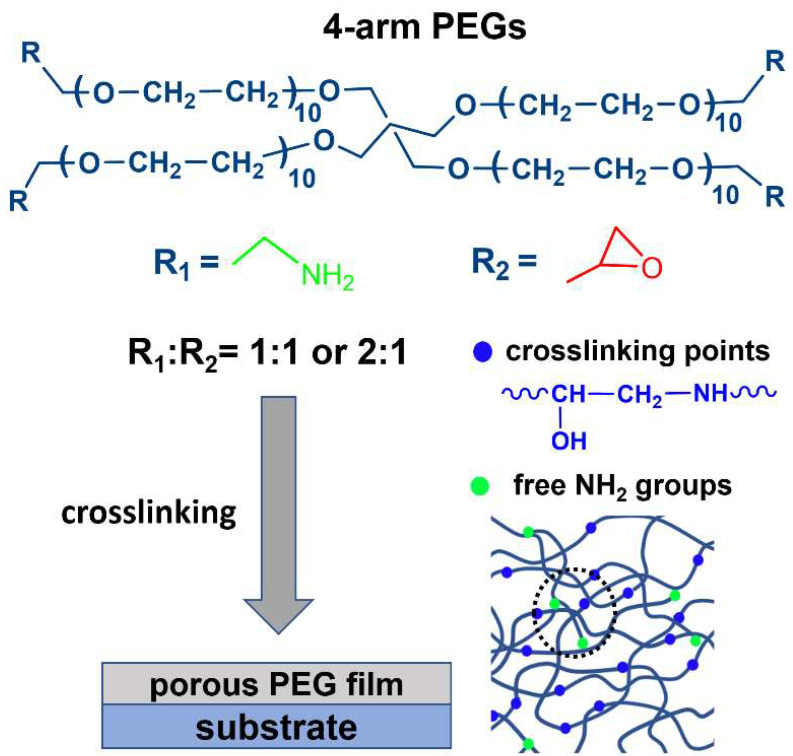
Structure of the STAR-NH_2_ (R_1_) and STAR-EPX (R_2_) precursors and a schematic drawing of the PEG film fabrication procedure relying on the extensive crosslinking of the precursors, mediated by the reactions between their terminal amine (R_1_) and epoxy (R_2_) groups. The resulting crosslinking points (ethanol-amine bridges) are marked by blue circles in the porous PEG film scheme (bottom, right); free (non-reacted) NH_2_ groups are depicted as green circles. The mixing ratio of the precursors was varied.

Along with OEG-decorated SAMs, all-PEG materials can also be used, such as PEG brushes and polymer films [27,28,29,30]. Alternatively, porous PEG and PEG-based films can be used, taking advantage of 3D immobilization of ssDNA in contrast to the standard 2D assemblies provided by SAM supports and usual OEG-based polymers [31,32,33,34]. In particular, such porous films can be efficiently formed by thermally activated crosslinking of multi-armed STAR-PEG precursors, decorated with amine (STAR-NH_2_) or epoxy (STAR-EPX) groups, which build ethanol-amine bridges between individual arms of the precursors upon the crosslinking (Figure 1) [35]. The porosity of the resulting films is then predominantly determined by the length of the PEG arms which are linked together in the joint center of each of the precursors [36]. The thickness of these films can be flexibly adjusted in a range from several to hundreds of nanometers by varying the concentration of the precursors in the primary solutions [35,36]. Distinct bioinert, hydrogel, and elastic properties of these films, existing also in the form of free-standing membranes, make them useful for further modification and processing as well as for a variety of applications [35,36,37,38,39,40,41,42]. Due to their distinguished bioinertness, these films can also serve as a bioinert matrix which can be decorated with bioreceptors and subsequently used for specific biosensing. This ability has indeed been demonstrated in the case of proteins, relying on the well-known biotin-avidin key-lock affinity [41]. With this achievement in mind, it is interesting and promising to explore whether such a strategy is also suitable for the immobilization and hybridization of ssDNA, which is the subject of the present study. For this purpose, we used the STAR-NH_2_ and STAR-EPX precursors with moderate molecular weights, adjusting their mixing ratio to optimize the immobilization efficiency of ssDNA, and applied X-ray photoelectron spectroscopy (XPS) and complementary electrochemical techniques to monitor the immobilization and hybridization processes. 

## 2. Materials and Methods

### 2.1. Chemicals 

The 4-arm STAR-NH_2_ and 4-arm STAR-EPX compounds (Figure 1) with a molecular weight of 2000 g/mol were purchased from Creative PEGWorks (Chapel Hill, NC, USA) and used as received. These compounds are characterized by low polydispersity and high purity, viz. 99% for STAR-NH_2_ and 98% for STAR-EPX in terms of amine and epoxy substitution, respectively. According to the molecular weight, the PEG arms of these compounds contain 10-11 EG monomers, corresponding to an arm length of 3.5−3.9 nm. Desalted ssDNA sequences were purchased from Metabion International AG (Munich, Germany). The first group of these sequences included unmodified thymine (T) and adenine (A) homo-oligonucleotides, viz’−T5−3’ (T5), 5’−T10−3’ (T10), 5’−A5−3’ (A5), and 5’−A10−3’ (A10). The second group included substituted homo-oligonucleotides, viz. N-hydroxy succinimide ester-C10−T5−3’ (NHS-T5) and 5’−N-hydroxy succinimide ester-C10−T10−3’ (NHS-T10). Please note that the NHS esters are reactive groups formed by carbodiimide-activation of carboxylate molecules. NHS-ester-labeled compounds react with primary amines under physiologic to slightly alkaline conditions (pH 7.2 to 9) to yield stable amide bonds after the release of the NHS group [43]. Consequently, decoration of ssDNA strands with NHS ester should be a reasonable strategy to immobilize these strands into the PEG matrix over the NH_2_ groups which did not participate in the crosslinking reaction and retained their reactivity. Other chemicals were purchased from Sigma-Aldrich.

### 2.2. Film Fabrication

The PEG films were prepared by the established protocol [35], schematically illustrated in Figure 1. Accordingly, the STAR-NH_2_ and STAR-EPX precursors were separately dissolved in chloroform with specific concentrations, mixed together in a ratio of 1:1 (V/V), spin-coated onto SiO_2_ passivated Si substrates (Siegert Wafer GmbH, Aachen, Germany), and crosslinked by thermal annealing (6 h, 80 °C). The resulting films were extensively rinsed with ethanol to remove possible weakly bound material. Two kinds of films were prepared. In the first case, the same concentration of the STAR-NH_2_ and STAR-EPX precursors in the primary solutions was used (1:1 ratio), set to either 2 mg/mL or 25 mg/mL to obtain either thin (~15 nm) or thick (~100 nm) films. In the second case, the concentrations of 20 mg/mL for STAR-NH_2_ and 10 mg/mL for STAR-EPX were used (2:1 ratio), to obtain PEG films (~80 nm thickness) with a noticeable amount of free NH_2_ groups, suitable for the reaction with the NHS ester groups of the substituted homo-oligonucleotides. For the sake of brevity, we will refer to these systems further in the manuscript as the 1:1 and 2:1 films, respectively. The 2:1 PEG films were also fabricated on evaporated Au(111) substrates (30 nm Au on Si(100); Georg-Albert PVD-Beschichtungen, Silz, Germany)—specifically for electrochemical measurements (see below). These films had similar thickness and similar properties as the films on the Si/SiO_2_ substrates. The stability and robustness of these films was in particular verified by the fabrication of large-area, free-standing PEG membranes which feature extreme elasticity [44].

### 2.3. ssDNA Immobilization and Hybridization

**Figure 2:**
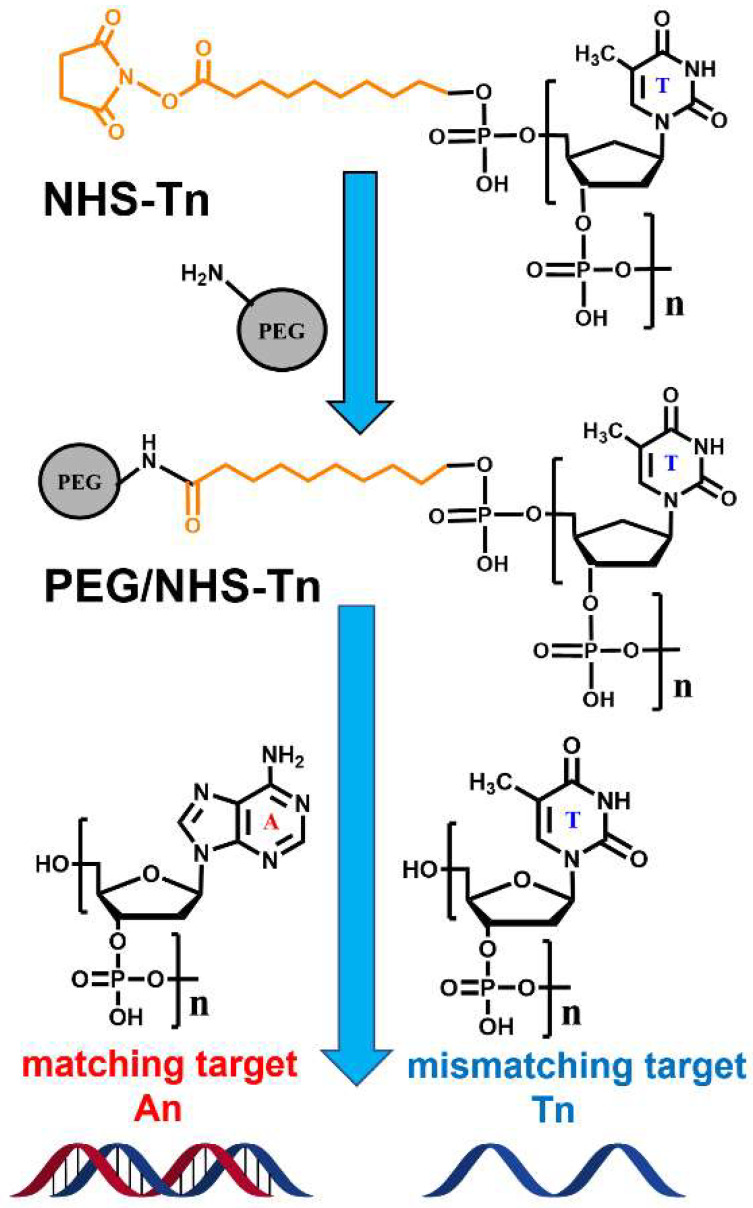
The structures of the NHS-Tn, An, and Tn compounds as well as a schematic illustration of the NHS-Tn immobilization in the PEG matrix (shown schematically as a gray circle) over the free (non-reacted) NH_2_ groups and its subsequent reaction with the An and Tn targets.

The procedures are schematically illustrated in Figure 2. For ssDNA immobilization, PEG films were immersed into 1M CaCl_2_ −TE buffer (10 mM Tris-HCl and 1 mM EDTA, pH = 7.4) containing 10 μM of ssDNA for 40 h at 37 °C. Please note that the 1M CaCl_2_-TE buffer was supplanted by PBS buffer (pH = 7.4) in the NHS-T5 and NHS-T10 cases to avoid reaction between the NHS ester group of ssDNA and the NH_2_ group of Tris-HCl. After the incubation, the samples were rinsed with Milli-Q water for 1 min and-dried under N_2_ flow.

For hybridization tests, the samples were immersed in a 1 M NaCl buffer containing 10 μM of the target sequences for 8 h at room temperature. After incubation, the samples were rinsed with 1 M NaCl buffer for 1 min, briefly dipped in a small amount of Milli-Q water (~0.5 mL) to remove excess salts, and finally dried with N_2_.

### 2.4. X-ray Photoelectron Spectroscopy

Bioinert properties of the PEG films, ssDNA immobilization in these films, and the hybridization ability of the resulting hybrid films were monitored by XPS, which is a frequently used technique for this purpose [5,45,46,47,48]. The measurements were performed using a MAX 200 (Leybold –Heraeus, Köln, Germany) spectrometer equipped with a hemispherical analyzer (EA 200; Leybold–Heraeus, Köln, Germany) and a Mg Kα X-ray source (260 W; ca. 1.5 cm distance to the samples). The spectra were obtained in normal emission geometry with an energy resolution of ~0.9 eV. The binding energy (BE) scale of the spectra was referenced to the Au 4f_7/2_ peak at 84.0 eV [49].

XPS was also used to estimate the areal density of the immobilized probe ssDNA. As a reference, we used a custom-designed SAM of nitrile-substituted naphthalenethiolates on Au(111) with an areal density of ~4.2 × 10^14^ molecules/cm^2^ [50], which in view of the molecular structure, is also the density of the terminal nitrogen atoms. Along with the intensities of the N 1s signals for the PEG-ssDNA and reference SAM, the difference in the number of the nitrogen atoms in the relevant molecules was taken into account.

### 2.5. Electrochemistry

Electrochemical measurements, which included cyclic voltammetry and electrochemical impedance spectroscopy (EIS), were performed using an IM6E potentiostat (Zahner-Elektrik GmbH & Co. KG, Kronach-Gundelsdorf, Germany) and a custom-made three-electrode electrochemical cell. Along with the working electrode (blank Au, Au/PEG, Au/PEG/ssDNA), an Ag/AgCl (non-aqueous) electrode and a platinum electrode (Osilla, Sheffield, UK) were used as the reference and counter electrodes, respectively. The blank Au substrates for the working electrode were purchased from Georg Albert, PVD-Beschichtungen (see also Section 2.1); the root mean square value of the surface roughness was estimated as ~0.5 nm as an average over the 0.5 × 0.5 μm^2^ and 5 × 5 μm^2^ scans (see Figure S1 in the Supporting Information). The cyclic voltammograms (CVs) and EIS data were recorded in a 10 mM Fe(CN)_6_^3−/4−^ electrolyte containing 0.1 M KCl. The exposed area of the electrodes was ~0.5 cm^2^. For CV measurements, a scan rate of 300 mV/s in the range from −0.8 V to +0.7 V (vs. Ag/AgCl) was applied. The EIS measurements were conducted at an alternating voltage with an amplitude of 5 mV, in the frequency range from 10^−1^ to 10^5^ Hz. Please note that an analogous approach was previously applied to monitor the performance of a ssDNA array immobilized on a glassy carbon electrode modified with a complex inorganic matrix on the basis of Sm_2_O_3_ nanoparticles and graphene oxide [51].

## 3. Results and Discussions

### 3.1. XPS

The monitoring of the relevant properties and processes within the given study relied on the well-known XP spectra of pristine PEG films and thymine and adenine homo-oligonucleotides. In accordance with the chemical composition, the PEG films are adequately represented by the C 1s, O 1s, and N 1s spectra [35,36,42], exhibiting the characteristic singular peaks at BEs of 286.8 eV (C 1s), 532.8 eV (O 1s), and 399.6 eV (N 1s), as shown in Figures S2 and S3 in the Supporting Information for the standard 1:1 case. The first two peaks are related to the PEG arms of the network and the third peak is representative of the nitrogen atoms in the ethanol-amine bridges. The homo-oligonucleotides can be best traced by the N 1s and P 2p spectra, representative of the nucleobases and phosphate groups in the ssDNA backbone, respectively. The P 2p spectra, usually showing a merged P 2p_3/2,1/2_ doublet at a BE of 133.5–133.7 eV [5,45,52,53,54], are not nucleobase-specific, but are a suitable fingerprint for the presence of ssDNA in the PEG matrix, which originally contains no phosphorus. The N 1s spectra are nucleobase-specific, which not only allows monitoring the presence of ssDNA but also allows to distinguish between thymine and adenine homo-oligonucleotides. In the case of thymine, the spectrum exhibits a single peak at a BE of ~400.5 eV, sometimes accompanied by a weak shoulder at a BE of ~398.5 eV associated with thymine moieties which are in direct contact with the substrate [5,52,55,56]. In the case of adenine, the spectrum consists of two peaks at BEs of ~398.7 and ~400.5 eV with the characteristic intensity ratio of 2:1 [5,52,56]. Significantly, the positions of these characteristic features do not change noticeably upon the T-A hybridization [56,57,58]. 

The PEG films are expected to be inert to ssDNA strands, similar to their behavior with respect to proteins [35,36]. To verify this assumption, 1:1 films were exposed to the unmodified ssDNA strands, viz. Tn and An (n = 5 and 10), and characterized by XPS. The respective C 1s, O 1s and N 1s XP spectra were found to be identical (within the experimental accuracy) to those of the original films (see Figure S2 in the Supporting Information), which demonstrates that the PEG films with the optimal mixing ratio of the precursors are indeed bioinert. 

These films contain, however, only a small amount of free amine groups (~3% according to our estimate, based on the infrared spectroscopy data from ref [35]), which can be insufficient for an effective immobilization of the NHS-ssDNA. Indeed, after the exposure of the 1:1 PEG films to NHS-T5 and NHS-T10, no noticeable changes could be observed in the XP spectra of the samples (see Figure S3 in the Supporting Information), indicating a very small (if at all) immobilization efficiency. As an additional proof, these samples were subsequently exposed to the target ssDNA strands complementary to T5 and T10, viz. A5 and A10, and characterized by XPS. Again, the spectra remained unchanged (see Figure S3 in the Supporting Information), which fully exclude that the probe T5 and T10 strands, capable of hybridizing with A5 and A10, were present in the PEG matrix. 

**Figure 3:**
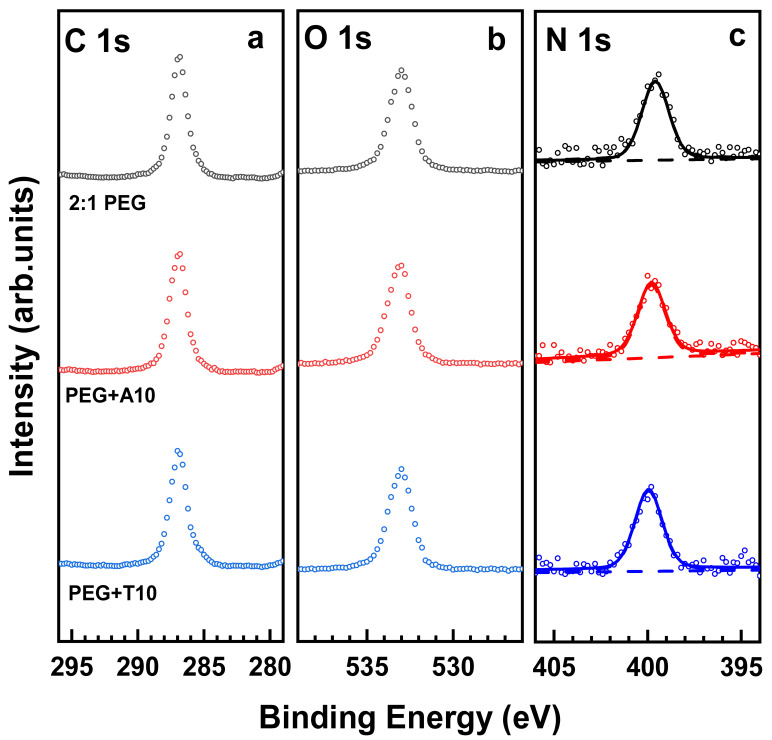
C 1s (**a**), O 1s (**b**) and N 1s (**c**) XP spectra of the 2:1 PEG films before (top curves) and after their incubation into the A10 and T10 solutions. The N 1s spectra are tentatively fitted by a single peak (solid lines) and a background (dashed lines).

The above results suggest that the 1:1 PEG films are not suitable for the immobilization of NHS-ester-modified ssDNA. A promising solution could then be a deviation from the 1:1 mixing ratio, resulting in a non-negligible amount of free amine groups, capable of reacting with the NHS ester moieties of the NHS-ssDNA. To verify this hypothesis, the STAR-NH_2_/STAR-EPX mixing ratio was set to 2:1 (see Section 2 for the experimental details) and 2:1 PEG films were prepared. Please note that the crosslinking reaction still works efficiently at even such a non-optimal mixing ratio, resulting in the formation of stable and robust PEG films, with the swelling and mechanical properties differing only slightly from those of the 1:1 films [41,44]. Additionally, the XP spectra of the 2:1 PEG films were found to be nearly identical to those of the 1:1 prototypes, with the characteristic C 1s, O 1s, and N 1s peaks at BEs of 286.8 eV, 532.8 eV, and 399.6 eV, respectively (Figure 3). The presence of only one N 1s peak in these spectra means that both crosslinked and free amine groups have nearly the same XPS binding energy. This circumstance simplifies the analysis of the spectra but make it difficult to provide an estimate for the amount of free amine groups.

For the next step, bioinert properties of the 2:1 PEG films were tested. For this purpose, these films were exposed to A10 and T10 and characterized afterwards by XPS, relying on the characteristic C 1s, O 1s, and N 1s spectra. The respective data are presented in Figure 3. The spectra of the films exposed to A10 and T10 turned out to be identical (within the experimental accuracy) to those of the original films. This observation means that a moderate deviation from the optimal mixing ratio does not result in a deterioration of bioinert properties. Thus, the 2:1 films can readily serve as a bioinert matrix for immobilization of probe ssDNA strands and subsequent hybridization with the target ssDNA strands, as far as immobilization and hybridization can be performed.

**Figure 4:**
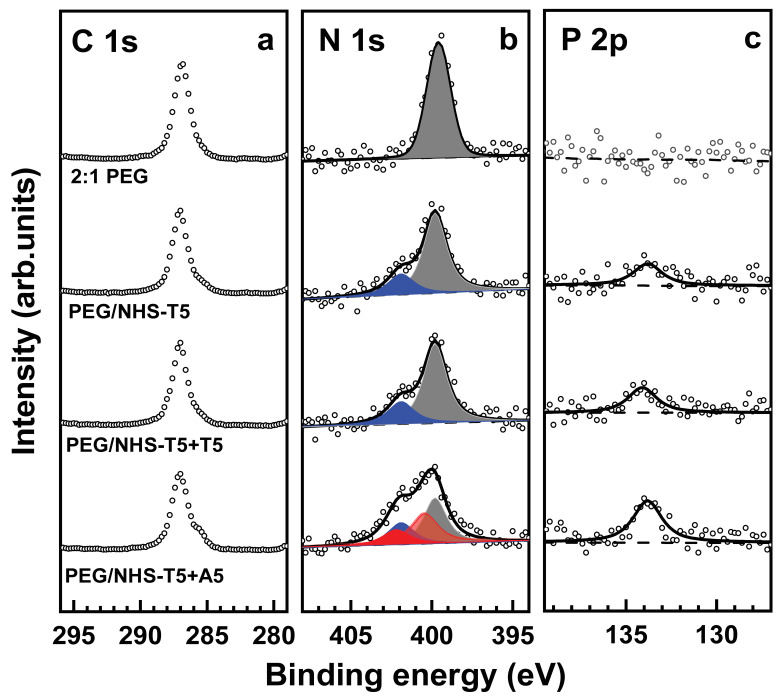
C 1s (**a**), N 1s (**b**), and P 2p (**c**) XP spectra of the original 2:1 PEG film, PEG film exposed to NHS-T5 (PEG/NHS-T5), and PEG/NHS-T5 probe film exposed to mismatching (T5) and matching (A5) target ssDNA. The N 1s spectra are decomposed into individual contributions related to the amine groups in the PEG matrix (dark gray), thymine (blue), and adenine (red). The P 2p spectra are tentatively fitted by a single peak (solid lines) and a linear background (dashed lines).

**Figure 5:**
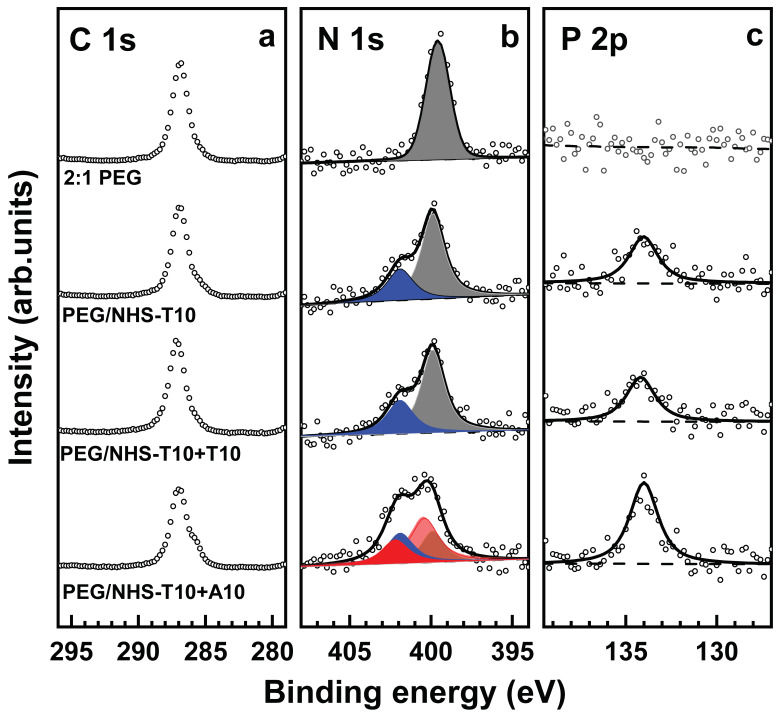
C 1s (**a**), N 1s (**b**), and P 2p (**c**) XP spectra of the original 2:1 PEG film, PEG film exposed to NHS-T10 (PEG/NHS-T10), and PEG/NHS-T10 probe film exposed to mismatching (T10) and matching (A10) target ssDNA. The N 1s spectra are decomposed into individual contributions related to the amine groups in the PEG matrix (dark gray), thymine (blue), and adenine (red). The P 2p spectra are tentatively fitted by a single peak (solid lines) and a linear background (dashed lines).

Both these processes turned out to be indeed possible. The immobilization of the probe ssDNA strands (T5 and T10) was carried out with the help of NHS-T5 and NHS-T10, relying on the reaction between the NHS ester group of the latter moieties and the free amine groups in the PEG films. The process was monitored by XPS, relying on the C 1s, N 1s and P 2p spectra. The respective data are shown in Figure 4 and Figure 5 for the NHS-T5 and NHS-T10 case, respectively. Let us first discuss the data for NHS-T5 and later - for NHS-T10.

After the exposure of the 2:1 PEG films to NHS-T5, the C 1s spectrum of the resulting films (PEG/NHS-T5) looks similar to that of the original PEG films, whereas the N 1s and P 2p spectra change noticeably. In the N 1s spectrum, the peak at ~399.8 eV, associated with the PEG matrix, decreases in intensity, and becomes accompanied by the characteristic peak of thymine at a BE of ~401.9 eV (see refs [5,52,55,56]). In the P 2p spectrum, a characteristic signature of the phosphate groups in the ssDNA skeleton at a BE of ~133.7 eV is observed [5,45,52,53,54]. This joint evidence indicates that the probe T5 strands were successively immobilized into the PEG matrix. 

Next, the ability of the T5-functionalized PEG films to probe a complimentary target ssDNA (A5) was tested by their incubation into A5 solution and subsequent characterization by XPS. Once again, the C 1s spectrum, representing predominantly the PEG matrix, did not change noticeably. In contrast, the N 1s and P 2p XP spectra, representing the ssDNA species, showed pronounced changes. In the P 2p spectrum, an increase in the intensity of the characteristic phosphate feature by a factor of ~1.78 is observed, corresponding to a high hybridization efficiency (~78%). In the N 1s spectrum, the shoulder at ~401.9 eV increases in intensity and becomes comparable to the main peak. Assuming that this increase stems from the T5-A5 hybridization, the spectrum was decomposed in three components associated with the NH_2_ and NH groups in the PEG matrix, thymine, and adenine. Within the respective fit, the PEG matrix was represented by a single peak at a BE of ~399.8 eV, thymine—by a single peak at 401.9 eV, and adenine—by two peaks at ~400.4 eV and ~402.2 eV with an intensity ratio of 2:1. As shown in Figure 4, the N 1 s spectrum could be fully reproduced by such a combination. The relative weights of the thymine and adenine components, corrected for the different contents of the nitrogen atoms in these bases (2 for thymine and 5 for adenine; see Figure 2), give then a hybridization efficiency of ~80%, in excellent agreement with the P 2p data.

To verify the selectivity of the T5-decorated PEG films to specific target, this film was exposed to a mismatching ssDNA sequence (T5) and examined by XPS. As shown in Figure 4, the C 1s, N 1s and P 2p XP spectra of the film taken before and after such an exposure are identical (within the experimental error), which indicates that the hybridization is indeed highly selective. 

The data for the immobilization of NHS-T10 into the 2:1 PEG films using NHS-T10 and the related hybridization tests with the matching (A10) and mismatching (T10) ssDNA sequence are presented in Figure 5. The same behavior as in the case of NHS-T5, A5, and T5 is observed (Figure 4). However, changes in the XP spectra upon the immobilization of the probe strands and their hybridization with the matching target are even more pronounced, which is understandable in view of the longer ssDNA chain and, subsequently, a larger spectral weight of the respective fingerprint features. Based on the decomposition of the N 1s spectra, the hybridization efficiency was estimated as ~89%, which is even somewhat higher than that for the shorter T5/A5 strands, driven, most likely, by a larger energy gain. The ssDNA-backbone-representative P 2p spectra, which show an intensity increase by a factor of ~1.88 upon the specific hybridization (T10-A10), give nearly the same value of the hybridization efficiency, supporting the reliability of the derived value. In contrast, similar to the T5/A5 case, no changes in the XP spectra were observed after the exposure of the T10-decorated PEG films to a mismatching sequence (T10).

The XPS data for the T5/A5 and T10/A10 series can also be compared to each other. In particular, both for the T10-decorated PEG films and the films subjected to the specific hybridization, the intensity of the P 2p signal is approximately double with respect to that in the T5/A5 case. This relation suggests a similar amount of the immobilized ssDNA species in the T10/A10 and T5/A5 cases. Such a behavior indicates that the ssDNA immobilization ability of the 2:1 PEG film does not depend strongly on the length of ssDNA strands but is predominantly determined by the amount of free amine groups. A tentative evaluation of the areal densities of the immobilized T5 and T10 probe strands, performed on the basis of the N 1s XP spectra and the nitrile-terminated SAM as a reference (see Section 2.4 for the technical details), gives the areal densities of 3.6 × 10^12^ strands/cm^2^ and 2.7 × 10^12^ strands/cm^2^ for the PEG/NHS-T5 and PEG/NHS-T10 assemblies, respectively. Note, however, that both these values represent coarse estimates only and are most likely somewhat higher in reality since the N 1s photoemission signal from the quasi-bulk PEG-ssDNA samples is diminished by self-attenuation, in contrast to the signal from the terminal nitrogen atoms of the reference SAM, which is not affected by the attenuation at all. 

The somewhat higher areal density for the PEG/NHS-T5 assembly compared to the PEG/NHS-T10 case is most likely related to a better permeability of the shorted NHS-T5 moieties in the PEG matrix. Nevertheless, the permeability is obviously still good enough for the NHS-T10 species, but can probably become a problem for noticeably longer ssDNA strands. Based on the length of the precursor arms (3.5–4 nm), a 3D PEG mesh with a characteristic pore size of 7–8 nm can be expected. This size is of course larger than the cross-sectional dimeter of ssDNA (~2 nm) but is, even for a short strand, much smaller than the ssDNA length, determined by the effective persistence length (~2 nm [59]) and the number of bases.

A related aspect is the behavior of the C 1s XP spectra. As was mentioned above and seen in Figure 4 and Figure 5, these spectra do not exhibit noticeable changes on the immobilization of the probe T5 and T10 strands into the PEG film, except probably a small decrease in intensity. This means that the signal of the PEG matrix, represented by a single peak at a BE of ~286.6 eV (see above), dominates over the signal of the ssDNA strands, overlapping partly with the PEG feature and represented by several peaks with specific intensity ratios and dominant spectral weight at a BE of 284.6–285.5 eV [45,60]. Consequently, and most likely, the immobilization of ssDNA does not involve the entire PEG film but, predominantly, the topmost part of it, occurring in a gradient fashion. Only after the specific hybridization, a small ssDNA-stemming shoulder at the low BE side of the PEG-related C 1s peak is observed. 

The permeability of ssDNA in the PEG matrix was additionally studied by exposure of comparably thin (15 nm) 1:1 PEG films to unmodified homo-oligonucleotides, T10 and A10. As demonstrated above, both 1:1 and 2:1 PEG films are generally inert to these biomolecules, so that any traces of T10 and A10 found in the spectra will most likely represent the strands penetrated through the film and adsorbed at the film-substrate interface, driven by their affinity to the non-bioinert Si/SiO_2_ substrate. Indeed, such traces could be found in the XP spectra of both PEG/T10 and PEG/A10 (Figure S4 in the Supporting Information), suggesting that the permeability depth of these strand into the PEG film is at least 15 nm. The affinity of the Si/SiO_2_ substrates to the ssDNA was additionally verified by their exposure to T10 and A10. The resulting XP spectra in Figure S5 (Supporting Information) show a noticeable increase in the intensity of the C 1s signal and appearance of the N 1s signal, which both indicate the adsorption of T10 and A10 onto the substrate. The C 1s spectra of both adsorbed ssDNA strands represent a single peak at a BE of 285.7–285.8 eV, accompanied by a weak high energy shoulder. Such spectra should indeed overlap significantly with the C 1s spectrum of the original PEG film, so that detection of ssDNA immobilization and hybridization on the basis of the C 1s XP spectra is hardly possible.

### 3.2. Electrochemical Studies 

The immobilization of the ssDNA into the PEG matrix and hybridization ability of the resulting assemblies were also monitored by electrochemical measurements, which were carried out for the 2:1 films only. These films were specifically fabricated on Au substrates serving as the working electrode in the electrochemical cell (see Section 2 for the technical details). Then, the recorded cyclic voltammograms (CVs) provided a measure of the electrochemical passivating ability of the PEG films (Au/PEG) and PEG/ssDNA assemblies (Au/PEG/ssDNA) toward the Fe(CN)_6_^3-/4-^ redox couples in the electrolyte solution. In contrast, the EIS analysis provided information on the charge transfer resistance (R_ct_) of the electrochemical cell.

**Figure 6:**
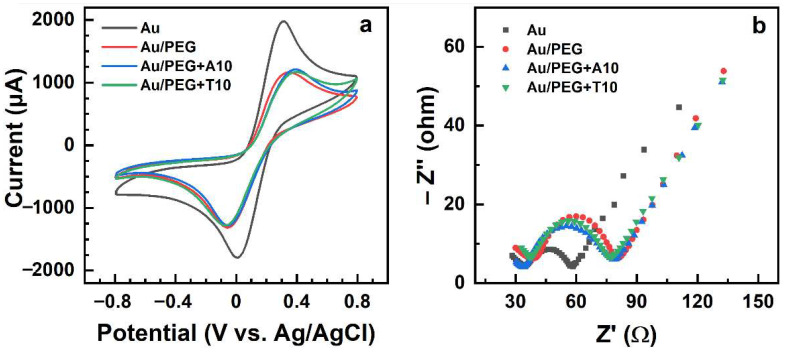
CVs (**a**) and Nyquist plots (**b**) for the blank Au electrode and Au/PEG electrode before and after its exposure to the unmodified ssDNA strands, A10 and T10.

As the first step, electrochemical passivating ability and bioinertness of the PEG films were tested. The respective data are shown in Figure 6 and the numerical results of the electrochemical measurements are summarized in Table 1. According to the CVs (Figure 6a) and Table 1, the presence of a ~80 nm PEG film on the Au electrode results in just a moderate suppression of the redox current and in just 33% decrease of the electrochemical capacitance, which is proportional to the area encircled by the respective CV [61]. Such a moderate reduction is most likely related to the porous structure of this film, which (structure) follows directly from the structure of the film precursors and the architecture of the PEG films (see Figure 1) as well as from the swelling and permeability properties of these films [35,36]. The porous structure is favorable for the efficient diffusion of the Fe(CN)_6_^3−/4−^ species toward the Au electrode. Additionally, the R_ct_ value did not change much after the introduction of the PEG film, increasing from 25 Ω to 40 Ω (Table 1), as follows from the Nyquist plots for the Au and Au/PEG samples in Figure 6b. The diameters of the semicircles in the high frequency region of these plots correspond to the R_ct_ values of the samples.

Exposure of the PEG films to the non-substituted ssDNA (A10 and T10) resulted in no obvious changes in their CVs (Figure 6a) and Nyquist plots (Figure 6b), with the nearly identical values of the relative capacitance and R_ct_ before and after exposure (Table 1). This behavior indicates the bioinert character of the 2:1 PEG matrix, in full agreement with the XPS data (see Section 3.1). 

**Figure 7:**
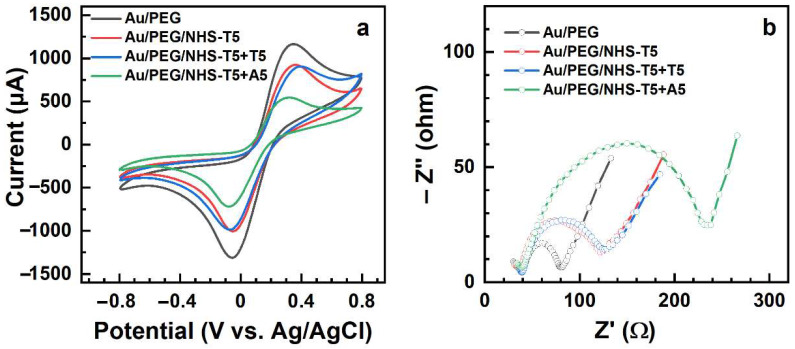
CVs (**a**) and Nyquist plots (**b**) for the Au/PEG working electrode and the Au/PEG/NHS-T5 electrode before and after its exposure to mismatching (T5) and matching (A5) target ssDNA.

**Figure 8:**
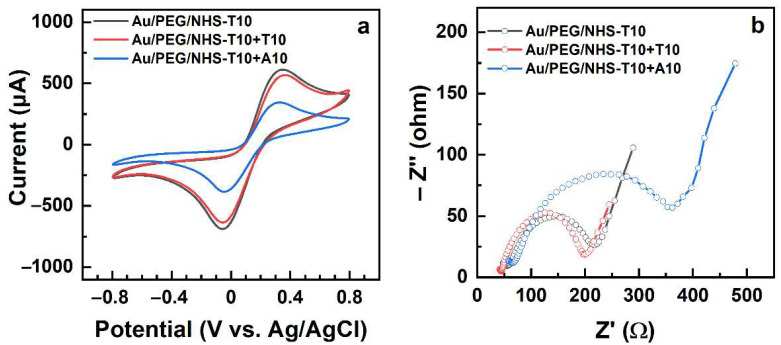
CVs (**a**) and Nyquist plots (**b**) for the Au/PEG/NHS-T10 working electrode before and after its exposure to mismatching (T10) and matching (A10) target ssDNA.

Subsequently, immobilization of NHS-T5 and NHS-T10 into the PEG matrix and the exposure of the resulting PEG-ssDNA films to the matching and non-matching target sequences was performed and the results were monitored by CV and EIS. The respective data are presented in Figure 7 and Figure 8; the derived values of the relative capacitance and R_ct_ are compiled in Table 1. Let us first discuss the data for NHS-T5 and later for NHS-T10. After the exposure of the Au/PEG to NHS-T5, the redox currents in the electrochemical cell decreased (Figure 7a), indicating a higher resistance of the working electrode. This effect is even more obvious in the Nyquist plots (Figure 7b), which show a noticeable increase in the diameter of the semicircle corresponding to an increase in R_ct_ from 41 Ω to 95 Ω (Table 1). This increase manifests the immobilization of the probe T5 strands into the PEG matrix and is explained by the effect of the negatively charged phosphate groups of the ssDNA, which hinder Fe(CN)_6_^3-/4-^ from diffusing to the electrode surface [51,62]. 

The exposure of the Au/PEG/NHS-T5 to the matching sequence (A5) resulted in further reduction of the redox current (Figure 7a) and relative capacitance (Table 1) as well as in a noticeable increase in the diameter of the semicircle in the Nyquist plots (Figure 7b), corresponding to a significant increase in R_ct_ from 95 Ω to 195 Ω (Table 1). This behavior manifests the efficient hybridization of T5 and A5, in full agreement with the XPS data (see Section 3.1). In contrast, the exposure of Au/PEG/NHS-T5 to the non-matching sequence (T5) resulted in only minor change in the CV (Figure 7a) and nearly no change in the Nyquist plot (Figure 7b) and R_ct_ value (97 Ω, Table 1). This behavior indicates that the hybridization did not occur for the mismatched sequence, again - in full agreement with the XPS data.

The electrochemical data for the immobilization of NHS-T10 into the PEG matrix and the subsequent exposure of the PEG/NHS-T10 probe to the mismatching (T10) and matching (A10) ssDNA sequences are shown in Figure 8. Both the CVs (Figure 8a) and the Nyquist plots (Figure 8b) exhibit the same behavior as the analogous data for the NHS-T5 case (Figure 7), which is also reflected by the relative capacitance and R_ct_ values in Table 1. In particular, the relative capacitance of Au/PEG decreased from 67% to 34% after the NHS-T10 exposure, while the R_ct_ value increased from 41 Ω to 158 Ω, manifesting the NHS-T10 immobilization in the PEG matrix. The exposure of the PEG/NHS-T10 probe to the matching sequence (A10) resulted in a further decrease of the relative capacitance from 34% to 19% and an increase of R_ct_ from 158 Ω to 330 Ω, manifesting a high hybridization efficiency. In contrast, no noticeable changes both in the experimental curves (Figure 8) and the derived fingerprint values (Table 1) were observed after the exposure of the PEG/NHS-T10 probe to the mismatching sequence (T10), manifesting thus a high selectivity of this probe.

Comparing the values for the NHS-T5 case with those for the NHS-T10 case in Table 1, viz. PEG/NHS-T5 vs PEG/NHS-T10 and PEG/NHS-T5+A5 vs PEG/NHS-T10+A10, we find that both the relative capacitance and R_ct_ do not reproduce exactly the factor of 2 describing the base number difference between T5/A5 and T10/A10. The observed relations can, on the one hand, be affected by the contributions from the PEG matrix and, on the other hand, reflect the somewhat different areal densities of the immobilized T5 and T10 moieties in the matrix. 

In contrast, both in the NHS-T5 and NHS-T10 case, the values of R_ct_ increase by a factor close to 2 after the hybridization with the matching A5 and A10 sequences, which means that R_ct_ can be used as a tentative measure of hybridization efficiency. Generally, looking at the data in Figure 6, Figure 7 and Figure 8, one can see that the Nyquist plots represent a much clearer and more distinct way to monitor the immobilization and hybridization processes in the PEG matrix than the CVs. Thus, EIS can be efficiently used as a transduction technique for these processes. 

**Figure 9:**
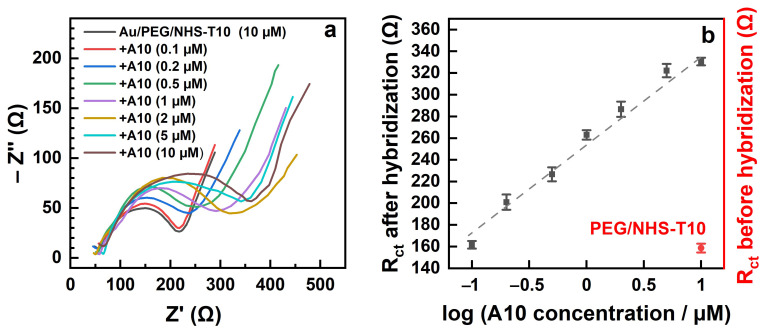
Nyquist plots (**a**) and the derived R_ct_ values (**b**) for the Au/PEG/NHS-T10 working electrode before (red symbol in **b**) and after (black symbols in b) its exposure to matching A10 target ssDNA. The concentration of A10 was varied. The R_ct_ behavior in (**b**) is tentatively traced by straight dashed line.

Finally, the sensitivity of this technique in the case of PEG/NHS-T10+A10 was tested. The concentration of A10 was varied from 10 μM (the standard value in this study; see Section 2.3) to 0.1 μM. The respective Nyquist plots are presented in Figure 9a and the derived values of R_ct_ are shown in Figure 9b. Accordingly and as expected, the R_ct_ value decreases progressively with the decreasing A10 concentration. This value is still noticeably higher than the reference value for PEG/NHS-T10 at 0.2 μM and nearly equal to the reference value at 0.1 μM. Consequently, the sensitivity of PEG/NHS-T10 to A10 is down to 0.1–0.2 μM, which can be probably improved even further by increasing the porosity of the PEG matrix and the areal density of the primary T10 probes.

## 4. Conclusions

In the present work, we fabricated a series of bioinert and porous PEG films, comprised of the cross-linked, amine-/epoxy-terminated STAR-PEGs, to explore their application as a platform for immobilization of probe ssDNA strands, capable of hybridization with complementary target sequences. As test ssDNA compounds, non-substituted and NHS-ester-substituted thymine and adenine homo-oligonucleotides were used, aiming for the immobilization of NHS-ester-modified strands by the reaction of the NHS ester groups with the non-reacted amine moieties in the PEG films. The immobilization and hybridization processes were monitored by XPS, relying on the ssDNA-specific P 2p and base-specific N 1s signals, as well as on electrochemical techniques, viz. cyclic voltammetry and electrochemical impedance spectroscopy. The results of the XPS and electrochemistry experiments agree completely with each other, underlying the reliability of the results.

It is demonstrated that the standard PEG films with a crosslinking-optimal precursor mixing ratio (STAR-NH_2_/STAR-EPX = 1:1) are not suitable for the NHS-ester-driven ssDNA immobilization, because of a very low concentration of the free (non-reacted) amine groups, used as coupling moieties for the NHS-ester-substituted ssDNA. To increase the amount of these groups, PEG films with the excess of the STAR-NH_2_ precursors were prepared (STAR-NH_2_/STAR-EPX = 2:1). These films showed the same complete inertness toward non-substituted ssDNA as the 1:1 layers but featured a much higher reactivity with respect to NHS-substituted ssDNA, allowing immobilizing probe ssDNA with a reasonable density. The films decorated with the probe ssDNA exhibited a high hybridization efficiency with respect to the matching target strands (78–89%), staying, at the same time, fully inert with respect to the non-matching ones. The efficiency was found to be somewhat higher for the longer strands compared to the shorter, presumably, because of a larger energy gain upon the hybridization. The most likely reason for the high hybridization efficiency is the 3D character of the probe ssDNA immobilization and sufficient separation of individual ssDNA probes in the PEG matrix, allowing their efficient accessibility by the target strands.

Both the immobilization and hybridization processes occurred predominately in the topmost part of the PEG films, which had a thickness of ca. 80 nm in most of the experiments. A significant reduction of this thickness should be avoided, since it results in the penetration of non-specific ssDNA strands to the substrate and their adhesion onto it, diminishing the bioinertness and specificity of the system. 

Whereas the monitoring of ssDNA hybridization by XPS requires a cost-intensive and complex equipment, electrochemical impedance spectroscopy, relying on a comparably simple and non-expensive setup, can be readily used as a label-free transduction technique in this context. This technique is also favorable compared to the standard experimental tools used in the field, as fluorescence spectroscopy (FS) and surface plasmon resonance (SPR), which either require DNA labeling (FS) or quite expensive equipment (SPR). In our case, the probe ssDNA-decorated PEG films can both be directly prepared on suitable electrodes and transferred onto them using the established film separation and transfer procedures [36,38,39,44]. The porous character of these films is of advantage for efficient diffusion of redox species in electrochemical cell, enabling reliable and to a certain extent even quantitative monitoring of hybridization. The ultimate sensitivity of the approach is reasonable and can be further improved at its practical implementation and optimization. Additionally, theoretical simulations can be helpful, since they provide a deeper insight in the mechanisms of surface reactions, also in relation to DNA [63,64,65,66].

## Figures and Tables

**Table 1:** Capacitance with Respect to the Cell with the Blank Au Working Electrode and the Charge Transfer Resistance Associated with the Specific Samples.

Sample	Relative Capacitance	Charge Transfer Resistance
Au	100%	25 Ω
Au/PEG	67%	41 Ω
Au/PEG+A10	65%	40 Ω
Au/PEG+T10	65%	43 Ω
Au/PEG/NHS-T5	50%	95 Ω
Au/PEG/NHS-T5+T5	49%	97 Ω
Au/PEG/NHS-T5+A5	32%	195 Ω
Au/PEG/NHS-T10	34%	158 Ω
Au/PEG/NHS-T10+T10	33%	155 Ω
Au/PEG/NHS-T10+A10	19%	330 Ω

## Data Availability

Data presented is contained within this paper. Additional data can be provided by the authors following a request.

## Supplementary Materials

The following supporting information can be downloaded at: https://www.mdpi.com/article/10.3390/bioengineering9090414/s1, Figure S1: Atomic force microscopy images of the working electrode, Figure S2: XP spectra of 1:1 PEG films at their incubation into the T5, A5, T10 and A10 solutions, Figure S3: XP spectra of 1:1 PEG films at their incubation into the T5 and T10 solutions and further incubation into matching target ssDNA solutions, Figure S4: XP spectra of the ultrathin 1:1 PEG film (15 nm) at their incubation into the A10 and T10 solutions, Figure S5: XP spectra of the Si/SiO_2_ substrate at their incubation into the A10 and T10 solutions.
