# Supplementary material for: Rational Design of Porous Poly(ethylene glycol) Films as a Matrix for ssDNA Immobilization and Hybridization
Source: Bioengineering (Basel). 2022 Aug 24;9(9):414. doi: 10.3390/bioengineering9090414 (PMC9496007; doi:10.3390/bioengineering9090414)
Supplement: Supplementary file 1 [file bioengineering-09-00414-s001.zip › bioengineering-1863522-supplementary.pdf]

Supporting Information

Rational Design of Porous Poly(ethylene glycol) Films as a Matrix for ssDNA Immobilization and Hybridization

Zhiyong Zhao, Saunak Das, and Michael Zharnikov*

Angewandte Physikalische Chemie, Universität Heidelberg, Im Neuenheimer Feld 253, 69120 Heidelberg, Germany

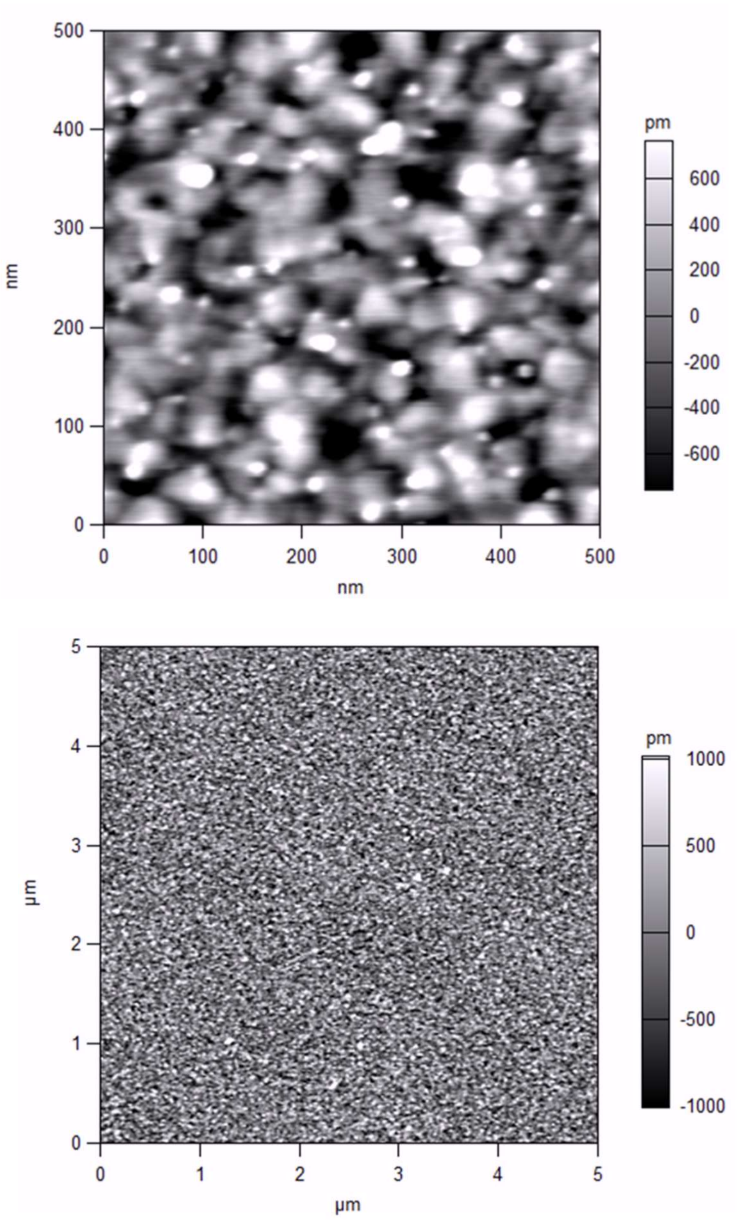

Figure S1. Atomic force microscopy images of the Au substrates used as the working electrode in the electrochemical experiments: $0.5 \times 0.5\ \mu m^2$ (top panel) and $5 \times 5\ \mu m^2$ (bottom panel) scans. For both scans, the root mean square value of the surface roughness was estimated as ~0.5 nm.

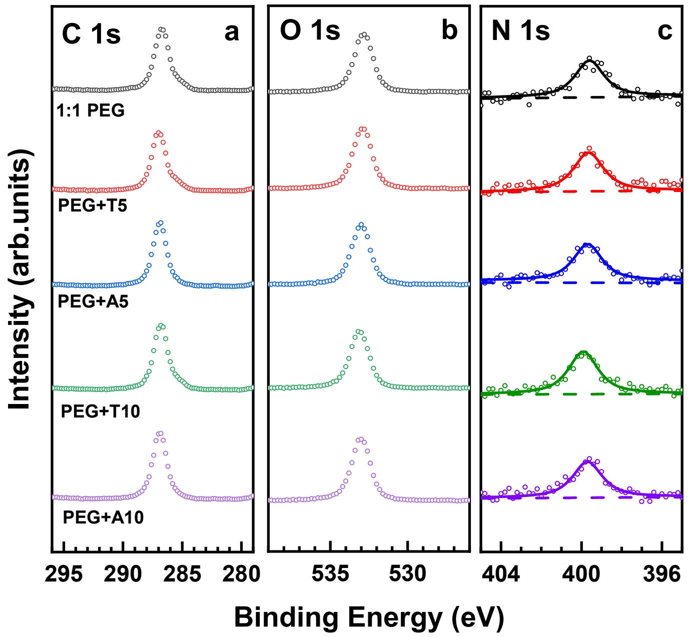

Figure S2. C 1s (a), O 1s (b) and N 1s (c) XP spectra of 1:1 PEG films before (top curves) and after their incubation into the T5, A5, T10 and A10 solutions.

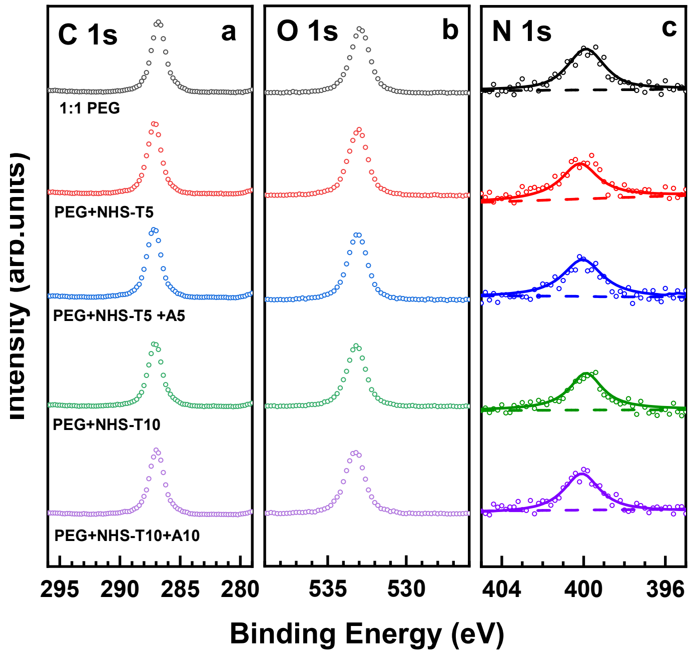

Figure S3. C 1s (a), O 1s (b) and N 1s XP spectra of 1:1 PEG films before (top curves) and after their incubation into the T5 and T10 solutions, and the corresponding films after once more incubation into matching target ssDNA solutions (A5 and A10).

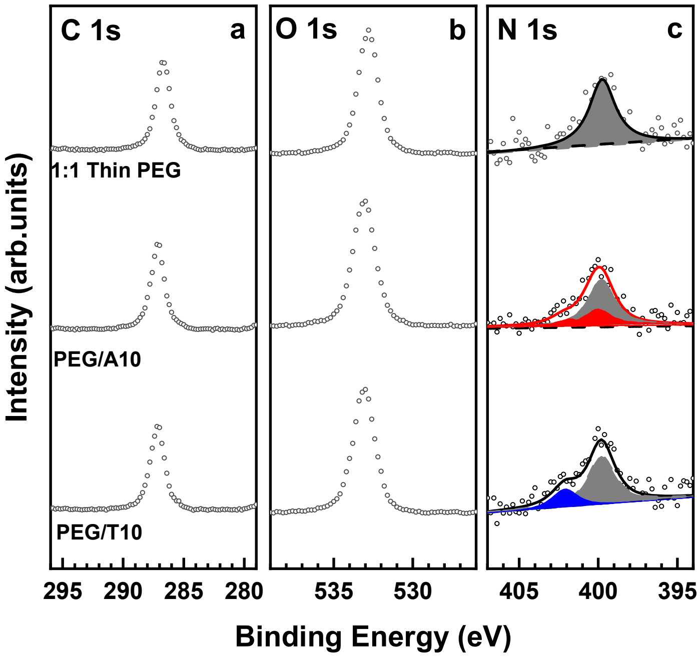

Figure S4. C 1s (a), O 1s (b) and N 1s XP spectra of the ultrathin 1:1 PEG film (15 nm) before and after their incubation into the A10 and T10 solutions. The N 1s spectra are decomposed into individual contributions related to the amine groups in the PEG matrix (dark gray), thymine (blue), and adenine (red).

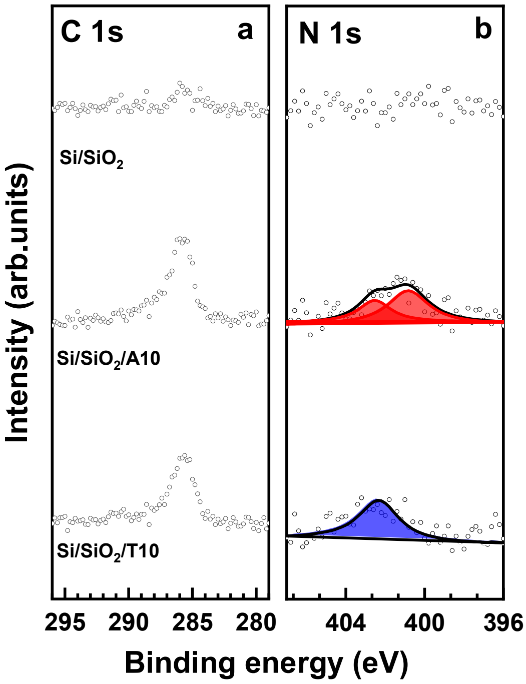

Figure S5. C 1s (a) and N1s (b) XP spectra of the Si/SiO_2 substrate before and after their incubation into the A10 and T10 solutions. The N 1s spectra are decomposed into individual contributions related to thymine (blue), and adenine (red).
